# Long-term neurotoxicity in paediatric patients exposed to general anesthesia: Is there a relationship between exposure to general anesthesia in children between 0 and 4 years of age and the subsequent development of ADHD in childhood?

**DOI:** 10.1192/j.eurpsy.2023.360

**Published:** 2023-07-19

**Authors:** B. Hernández Gajate, T. Gutiérrez Higueras, R. M. Fiestas Velasco, V. Rubio de la Rubia, F. Calera Cortés

**Affiliations:** Psychiatry, Hospital Universitario Reina Sofía, Córdoba, Spain

## Abstract

**Introduction:**

The Food and Drug Administration (FDA) recently issued new warnings about the possible effects of the repeated or prolonged use of general anaesthesia and sedatives on the brain development of children under 4 years old during surgeries or paediatric procedures.

**Objectives:**

To evaluate the possible long-term neurotoxic impact the exposure to general anaesthesia has on the paediatric population from 0 to 4 years, which is the period during which the brain develops.

**Methods:**

Initially, a search for observational studies that described the risk of neurotoxicity and alterations in the long-term cognitive development of children exposed to general anaesthesia before 4 years of age, was performed in PubMed between 2016 and 2020.

**Results:**

Finally, 5 retrospective cohort studies comparing children exposed and not exposed to general anaesthesia were included in this study. None of these showed significant differences in their main study variables. However, three of this studies found significant differences in some of the secondary variables such as speed of processing, motor skills, internalization of behaviour and learning, and attention deficit hyperactivity disorder (ADHD).

**Conclusions:**

In vitro and in vivo studies of anesthetics have shown serious neurotoxic effects in the developing brain. However, the clinical relevance of these findings for children undergoing anesthesia remains unclear.

Most of these studies suggest a strong relationship between exposure to anesthesia in children aged 0 to 4 years, this being greater after multiple exposures. Despite these results, many of these articles conclude that further research is needed on this topic.

**Disclosure of Interest:**

None Declared

